# The impact of single and pairwise Toll-like receptor activation on neuroinflammation and neurodegeneration

**DOI:** 10.1186/s12974-014-0166-7

**Published:** 2014-09-20

**Authors:** Karen Rosenberger, Katja Derkow, Paul Dembny, Christina Krüger, Eckart Schott, Seija Lehnardt

**Affiliations:** Department of Neurology, Charité-Universitaetsmedizin Berlin, Charitéplatz 1, 10117 Berlin, Germany; Department of Hepatology and Gastroenterology, Charité-Universitaetsmedizin Berlin, Augustenburger Platz 1, 13353 Berlin, Germany; Cluster of Excellence NeuroCure, Charité-Universitaetsmedizin Berlin, Charitéplatz 1, 10117 Berlin, Germany; Institute of Cell Biology and Neurobiology, Center for Anatomy, Charité-Universitaetsmedizin Berlin, Charitéplatz 1, 10117 Berlin, Germany

**Keywords:** Chemokines, Cytokines, Toll-like receptors, Microglia, Neurodegeneration, Neuroinflammation, Pairwise TLR activation

## Abstract

**Background:**

Toll-like receptors (TLRs) enable innate immune cells to respond to pathogen- and host-derived molecules. The central nervous system (CNS) exhibits most of the TLRs identified with predominant expression in microglia, the major immune cells of the brain. Although individual TLRs have been shown to contribute to CNS disorders, the consequences of multiple activated TLRs on the brain are unclear. We therefore systematically investigated and compared the impact of sole and pairwise TLR activation on CNS inflammation and injury.

**Methods:**

Selected TLRs expressed in microglia and neurons were stimulated with their specific TLR ligands in varying combinations. Cell cultures were then analyzed by immunocytochemistry, FlowCytomix, and ELISA. To determine neuronal injury and neuroinflammation *in vivo*, C57BL/6J mice were injected intrathecally with TLR agonists. Subsequently, brain sections were analyzed by quantitative real-time PCR and immunohistochemistry.

**Results:**

Simultaneous stimulation of TLR4 plus TLR2, TLR4 plus TLR9, and TLR2 plus TLR9 in microglia by their respective specific ligands results in an increased inflammatory response compared to activation of the respective single TLR *in vitro*. In contrast, additional activation of TLR7 suppresses the inflammatory response mediated by the respective ligands for TLR2, TLR4, or TLR9 up to 24 h, indicating that specific combinations of activated TLRs individually modulate the inflammatory response. Accordingly, the composition of the inflammatory response pattern generated by microglia varies depending on the identity and combination of the activated TLRs engaged. Likewise, neuronal injury occurs in response to activation of only selected TLRs and TLR combinations *in vitro*. Activation of TLR2, TLR4, TLR7, and TLR9 in the brain by intrathecal injection of the respective TLR ligand into C57BL/6J mice leads to specific expression patterns of distinct TLR mRNAs in the brain and causes influx of leukocytes and inflammatory mediators into the cerebrospinal fluid to a variable extent. Also, the intensity of the inflammatory response and neurodegenerative effects differs according to the respective activated TLR and TLR combinations used *in vivo.*

**Conclusions:**

Sole and pairwise activation of TLRs modifies the pattern and extent of inflammation and neurodegeneration in the CNS, thereby enabling innate immunity to take account of the CNS diseases’ diversity.

**Electronic supplementary material:**

The online version of this article (doi:10.1186/s12974-014-0166-7) contains supplementary material, which is available to authorized users.

## Background

The family of Toll-like receptors (TLRs) comprises germ-line encoded pathogen recognition receptors that allow the innate immune system to differentiate among microorganisms by sensing their conserved motifs called pathogen-associated molecular patterns [1]. Although signaling pathways are shared by different TLRs, several factors constitute the complexity of TLR function and outcome. First, the subcellular distribution of the receptors differs. Whereas TLRs 1, 2, and 4 to 6 primarily, but not exclusively, localize to the plasma membrane, the class of nucleotide sensing TLRs 3 and 7 to 9 are primarily associated with intracellular vesicular compartments such as endosomes [2]. Secondly, TLRs modulate antigen-specificity by forming homo- and heterodimers (e.g., TLR1/2, TLR2/6) and by adapting co-receptors and binding proteins (e.g., TLR4/CD14/MD2). Thirdly, TLRs are activated by a myriad of diverse agonists. Intracellular TLRs sense nucleic acid-based agonists, such as dsRNA, ssRNA, and unmethylated cytosine-phosphate-guanine motifs (CpG ODN), thereby being particularly specialized in viral recognition. In contrast, TLRs expressed on the cell surface detect lipopeptides, glycolipids, and flagellin derived from a large variety of organisms including bacteria, parasites, and fungi. Finally, engagement of TLRs by their cognate ligands leads to the recruitment of one or more of five intracellular adaptor proteins, including myeloid differentiation primary response gene 88 (MyD88) and TIR-domain-containing adaptor inducing IFN-β (TRIF). MyD88 is required for all TLR signaling pathways except TLR3 and a TLR4/MyD88-independent pathway. One major TLR-induced set of responses is the activation of transcription factors, such as NF-κB, leading to the induction of proinflammatory mediators and type I interferons [1].

TLRs do not only recognize pathogen-associated molecular patterns (PAMPs) but also host-derived damage-associated molecular patterns (DAMPs). DAMPs identified as TLR ligands include heat shock protein 60 (HSP60) and fibrinogen for TLR4 as well as gangliosides and hyaluronic acid fragments for TLR2 [3]. Most of these molecules are derived from extracellular matrix or are released by injured cells, making them accessible to TLRs, and thereby provoke inflammation during pathologies. This setting may contribute to both infectious and non-infectious central nervous system (CNS) injury [4].

All cells of the CNS express TLRs, while each cell type displays its own subset of differentially expressed TLRs and related signaling adaptor proteins [5-10]. The dynamic expression of TLRs, some of which appears on certain cell types only in specific settings, implicates their participation and functional relevance in both physiological and pathological states of the CNS [9,11,12]. In particular, TLRs and associated signaling pathways contribute to various CNS diseases such as infection, stroke, classical neurodegenerative diseases, and multiple sclerosis [13,14]. In general, CNS disorders are characterized by inflammation and neuronal injury. Inflammation and tissue damage exert the beneficial effect of clearance of pathogens, cell debris, and other harmful components released from injured cells thereby restoring homeostasis and enabling repair. However, exaggerated activation of innate immunity in the brain can cause further tissue damage, thereby exacerbating the initial brain insult [15,16]. Activation of TLRs in microglia, the major players in the innate immune surveillance within the brain, results in secretion of neurotoxic molecules such as cytokines and reactive oxygen species *in vitro* [17,18]. The concept of receptor redundancy for an effective innate immune response is still comprehensible, especially in the brain, given the potential for invading pathogens or cellular disturbances to cause significant damage in a tissue with only limited regenerative capacity.

It is conceivable that in a specific setting within a physiological or pathological context in the CNS, more than one TLR ligand is present and multiple TLRs are activated simultaneously at one time. As the various TLRs and TLR complexes may trigger specific intracellular pathways, the signal resulting from the activation of a specific TLR or a specific combination of TLRs may induce a response individually suited for the respective cause of TLR activation. To systematically investigate and compare the impact of sole TLR activation and combined TLR activation on CNS inflammation and injury, we stimulated selected TLRs expressed in microglia and neurons with their highly specific pathogen-associated and host-derived ligands in varying combinations *in vitro* and *in vivo*. Our results indicate that the extent and pattern of the subsequent inflammatory response and neuronal injury in the CNS varies specifically according to the activated TLR and the TLR combination engaged.

## Methods

### Animals

C57BL/6J mice were purchased from Charles River, Sulzbach, Germany. TLR4 knock out (TLR4KO) mice were generously provided by Dr. S. Akira (Osaka University, Department of Host Defense, Osaka, Japan). All animals were maintained according to the guidelines of the committee for animal care. Experimental procedures were performed in accordance with the institutional review committee Landesamt für Gesundheit und Soziales, Berlin.

### TLR ligands

Lipopolysaccharide (LPS), Pam3CysSK4, loxoribine, imiquimod, and CpG ODN were obtained from Invivogen, San Diego, USA. HSP60 was obtained as low-endotoxin charge from Enzo Life Sciences, Lörrach, Germany. Dose response studies with the different ligands were performed in microglial and neuronal cultures analyzed by TNF-α ELISA (Additional file 1: Figure S1A) and by quantification of NeuN^+^ cells per field (Additional file 1: Figure S1B), respectively. The concentrations of the respective ligands that induce maximum responses were in part published in previous work [7,18,19] and were used in this study.

### Primary culture of purified microglia

Purified microglia were generated from forebrains of 0- to 3-day-old mice as described previously [20]. For analysis of the inflammatory response *in vitro*, microglia were plated at 3 × 10^4^ cells/96-well.

### Primary cultures of neurons and co-cultures of neurons and microglia

Purified neurons were generated from mouse embryos at gestational stage 17 as described previously [20]. Cells were plated on glass cover slips treated with poly-d-lysine at 5 × 10^5^ cells/24-well in 500 μL. After 24 h, half of the medium was replaced with fresh pre-warmed medium. Purity of cultures was at least 96% after plating as verified by immunostaining with NeuN and glial fibrillary acidic protein (GFAP) antibodies (both obtained from Chemicon, Temecula, USA) and IB4 (Invitrogen, Carlsbad, USA). For co-cultures, microglia were added to DIV3-neurons at 6 to 7.5 × 10^4^ cells/24-well in 500 μL.

### TNF ELISA

TNF-α concentrations in cell culture supernatants were measured by enzyme-linked immunosorbent assay (ELISA) using the BD OptEIA™ Set Mouse TNF (BD Biosciences, San Diego, USA) according to the manufacturer’s manual.

### Multiple analyte detection

Multiple analyte detection of cytokines and chemokines in supernatants and cerebrospinal fluid (CSF) was performed using FlowCytomix (Bender MedSytems, Burlingame, USA). The immunoassay is a bead-based method to detect the concentrations of up to 20 analytes in one sample using a flow cytometer. The mouse/rat basic kit was used in combination with mouse simplex kits. Inflammatory mediators analyzed by FlowCytomix assay included TNF-α, CXCL1, CCL2, CCL5, IL-6, IL-10, IL-4, IL-17, IFN-γ, IL-12p70, IL-13, IL-1α, GM-CSF, and IL-23.

### Analysis of nitric oxide (NO) content

The nitric oxide (NO) content was measured indirectly by assaying the stable end product nitrite with the standard Griess reaction, as previously described [18].

### Quantification of CNS cells *in vitro*

Co-cultures were quantified 3 days after starting the respective incubation. For analysis of neuronal survival, cell cultures were immunostained with NeuN antibody. NeuN^+^ cells were quantified in 6 to 10 fields (at 600× magnification) per coverslip, and the mean was calculated. Data are expressed as relative to control (=100%). Caspase-3-positive cells (activated caspase-3 Ab, Millipore, Darmstadt, Germany) were quantified in 6 to 10 fields (at 100× magnification) per coverslip, and the mean was calculated. All quantifications were performed in a blinded manner.

### Intrathecal injection into mice

Intrathecal injection into mice and analysis of the CSF were performed as described previously [7,16,19]. In detail, 40 μL of ligand solution (PCR studies after 12 h: 10 μg LPS, 10 μg Pam3CysSK4, 10 μg imiquimod, 10 μg CpG ODN; histologic studies after 72 h: 10 μg LPS, 40 μg Pam3CysSK4, 136 μg loxoribine, 10 μg CpG ODN, all obtained from Invivogen, or 10 μg HSP60 (Enzo Life Sciences) were injected intrathecally into 6- to 8-week-old male mice. Notably, the contamination with LPS of the HSP60 preparation as declared by the manufacturer was <50 EU/mg and ≤1.67 EU/mg, as determined by an independent laboratory specializing in endotoxin testing and published in previous work (Mikrobiologisches Labor, Münster, Germany; see also [18]). For real-time PCR analysis, brains were removed and cut along the sulcus medianus into two halves, which were separately snap-frozen in liquid nitrogen. For immunohistochemical studies, mice were perfused transcardially with isotonic-saline followed by 4% paraformaldehyde (PFA). Brains were removed, subjected to a row of 10%, 20%, and finally 30% sucrose in 0.1 M phosphate buffered saline (PBS) for cryoprotection, frozen in 2-methylbutan on dry ice, and stored at –80°C until sectioning.

### Real-time PCR

One brain hemisphere was homogenized in 1 mL TRIzol® (Invitrogen, Darmstadt, Germany) with an Ultra-Turrax® at 21,500 rpm for 30 sec. The homogenate was centrifuged at 12,000× *g* and 4°C for 15 min. DNA was removed with RQ1 RNase-free DNase (Promega, Madison, USA) and UltraPure™ phenol:chloroform:isoamyl alcohol (Invitrogen). For synthesis of cDNA from 1 μg RNA, random hexamers (Roche, Mannheim, Germany) were used with MMLV-RT (Promega). SYBR® Green-based quantitative real-time PCR was performed with the RT^2^ qPCR Primer Assays (SABiosciences Corporation, Frederick, USA) according to the manufacturer’s manual with the RT^2^ Real-Time™ PCR protocol (#1) and the ABI7500 default dissociation stage. Glyceraldehyde-3-phosphate dehydrogenase was used as a housekeeping gene due to the fact that its expression was not influenced by treatments. For statistics, dCT values (CT_gene of interest_ – CT_HKG_) were log2-transformed according to [21]. Fold exchange (2^-(ddCT)^) of treated mice relative to control was calculated using the median of each group.

### Immunohistochemistry

Coronal brain sections of five representative levels (interaural 6.60 mm, 5.34 mm, 3.94 mm, 1.86 mm, and –0.08 mm) were fixed with 4% PFA, washed with PBS, and treated with blocking solution (PBS + 0.1% Triton-X 100 + 5% normal goat serum) for 1 h. They were then incubated with the primary antibody (anti-NeuN, anti-neurofilament, anti-GFAP, all purchased from Chemicon, Temecula, USA; Iba1 purchased from WAKO, Richmond, USA; activated caspase-3 Ab, Millipore, Darmstadt, Germany) at 1:1,000 overnight at 4°C. Subsequently, sections were incubated with the relevant secondary antibody (all purchased from Jackson Immuno Research, West Grove, USA) for 1 h at room temperature. Nuclei were stained with DAPI.

### Quantification of CNS cells *in vivo*

Cells in brain sections were quantified 3 days after the respective intrathecal injection into mice. For analysis of neuronal survival sections were stained with NeuN antibody or an antibody against activated caspase-3. NeuN^+^ or caspase-3^+^ cells were counted in three fields (at 600× magnification) of the cerebral cortex per hemisphere at interaural 1.86 mm, and the mean was calculated, which is expressed as NeuN^+^ or caspase-3^+^ cells per mm^2^. For further analysis of apoptotic cells, sections were stained by DAPI. Glial cells were quantified by staining brain sections with Iba1 or GFAP antibodies to mark microglia and astrocytes, respectively. Iba1- and GFAP-positive cells were quantified and calculated as described above. All quantifications were performed in a blinded manner.

### Statistical analysis

Data are expressed as indicated in the figures. Statistics as indicated in the figure legends were calculated using GraphPad Prism version 5.01 for Windows (GraphPad Software, San Diego, USA). Differences were considered statistically significant when *P* <0.05.

## Results

### Pairwise stimulation of TLRs induces an inflammatory response in microglia that differs from sole TLR activation *in vitro*

In immune cells, stimulation of TLRs with their respective specific ligands initiates the canonical signaling pathway, which results in activation of transcription factors, including NF-κB, and ultimately leads to the secretion of proinflammatory molecules [1]. Microglia constitutively express mRNA of most of the TLRs identified so far [22]. Since it is well established that microglia readily release TNF-α upon TLR stimulation [16,23], we used the analysis of TNF-α production as a functional assay to elucidate the inflammatory response in microglia induced by single versus combined TLR stimulation. To this end, microglia isolated from C57BL/6J mice were incubated with LPS as a highly specific ligand for TLR4, Pam3CysSK4 as a specific TLR2 agonist acting mainly through TLR2/1 heterodimeric receptors, loxoribine as a ligand for TLR7, or CpG ODN as a TLR9-specific agonist solely or in pairwise combination, as indicated, for up to 72 h. The concentrations of the ligands were based on dose response experiments in microglia and neurons (see *Methods* section) and comply with well-established working doses described in other studies [24-27].

**Figure 1:**
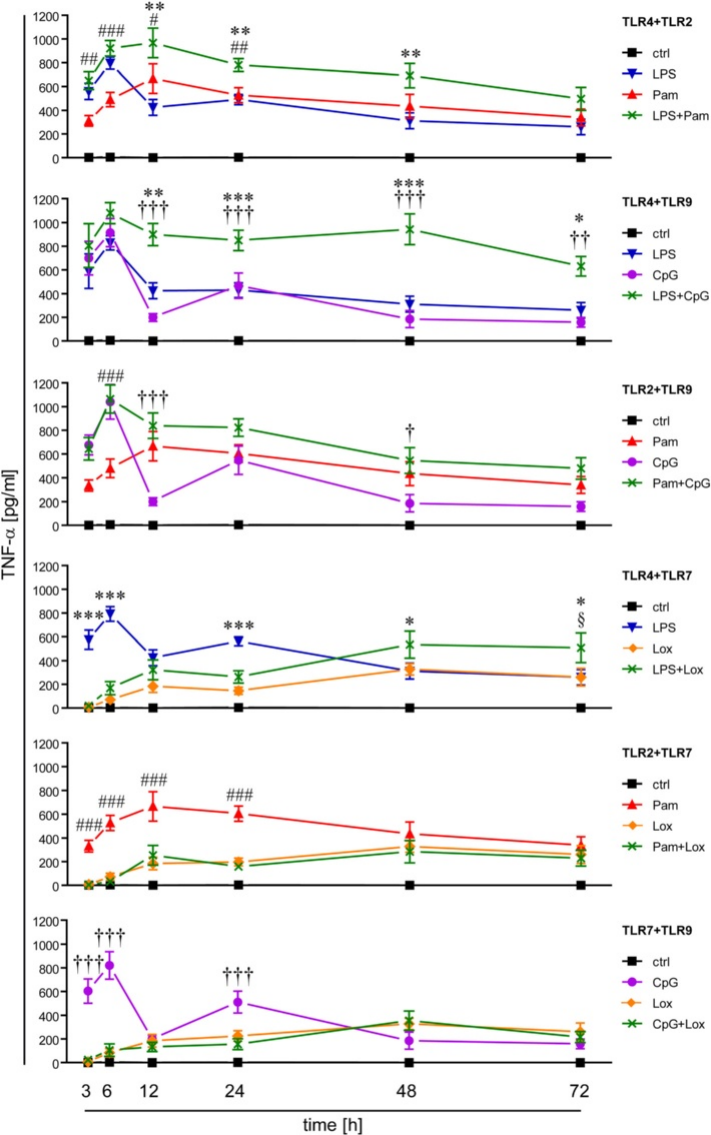
**Activation of single and pairwise TLRs results in a distinct inflammatory response in microglia.** Purified microglia were stimulated with LPS (100 ng/mL), Pam3CysSK4 (Pam, 100 ng/mL), loxoribine (lox, 1 mM), or CpG ODN (CpG, 1 μM) alone or simultaneously with pairwise combinations of the ligands, as indicated. PBS served as control. Supernatants were collected at indicated time points and analyzed by TNF-α ELISA; mean ± SEM of five independent experiments run with duplicates. Two-way ANOVA with Bonferroni-selected pairs of each individual compound vs. combination of compounds (*LPS vs. ligand combination; #Pam vs. ligand combination; †CpG vs. ligand combination; §lox vs. ligand combination). P*, #, †, § <0.05; P**, ##, ††, §§ <0.005; P***, ###, †††, §§§ <0.001.

Supernatants of microglial cultures were collected at indicated time points and subjected to ELISA to measure TNF-α concentrations (Figure 1). Each of the four TLR ligands named above induced secretion of TNF-α from microglia. However, a direct comparison between the TNF-α levels induced by the different TLRs is not feasible due to the differing properties and concentrations of the various ligands. TNF-α amounts in supernatants of unchallenged microglia were low or undetectable during the whole round of observation (Figure 1). Simultaneous challenge of microglia with LPS plus Pam3CysSK4, thereby activating TLR4 and TLR2, respectively, led to an additive increase in TNF-α release from 3 h on to 72 h, which was significant compared to both single application of LPS and Pam3CysSK4 at 12 h and 24 h. Simultaneous stimulation of microglia with LPS plus CpG ODN, thereby activating TLR4 and TLR9, respectively, induced significant additive levels of TNF-α from 12 h to 72 h compared to sole stimulation of TLR4 or TLR9. The combination of Pam3CysSK4 plus CpG ODN, thereby activating TLR2 and TLR9, respectively, resulted in a slight increase of TNF-α amounts compared to sole stimulation of TLR2 or TLR9 (Figure 1). TNF-α levels in response to simultaneous stimulation with two different ligands remained higher than levels after stimulation with single ligands even when double doses of the respective individual TLR ligand were used (data not shown). Thus, simultaneous stimulation of TLR2 plus TLR4, TLR4 plus TLR9, and to a lesser extent TLR2 plus TLR9, which all belong to the bacteria sensing TLR family, resulted in prolonged elevated TNF-α secretion compared with the stimulation of the respective single receptor. In contrast, activation of the virus sensing TLR7 with loxoribine additionally to the stimulation with LPS, Pam3CysSK4, or CpG ODN suppressed TNF-α secretion induced by sole stimulation with the respective ligand to levels induced by loxoribine alone from 3 h on to 24 h. However, at 72 h, simultaneous stimulation of TLR4 and TLR7 induced significant additive levels of TNF-α compared to sole stimulation of TLR4 or TLR7 (Figure 1). Similar results were obtained using imiquimod as a further TLR7-specific ligand (data not shown).

To test whether activation of TLR3 (Additional file 2: Figure S2A) exerts similar inhibitory effects on TNF-α secretion induced by other TLR ligands as was observed for TLR7, microglia were incubated with the respective ligands named above alone or in combination with poly(I:C), a TLR3-specific ligand, or loxoribine. Resulting supernatants were analyzed by ELISA for TNF-α secretion after 6 h (Additional file 2: Figure S2B). Additional treatment with poly(I:C) did not affect the TNF-α response induced by Pam3CysSK4, LPS, or CpG ODN alone, while additional treatment with loxoribine suppressed these responses, as expected. Further studies revealed that supernatants of microglia incubated with loxoribine did not contain significant amounts of interferon (IFN)-α, a major type I IFN, which can be induced by activation of TLR7 in peripheral immune cells [1] (data not shown). In line with these findings, inhibitory effects mediated by TLR7 activation were not affected by the presence of an inhibitory antibody against IFN-α/β receptor I (data not shown).

In summary, stimulation of a single TLR and simultaneous stimulation of two TLRs result in distinct inflammatory responses in microglia.

### Simultaneous activation of two TLRs results in a distinct cytokine and chemokine profile in microglia

**Figure 2:**
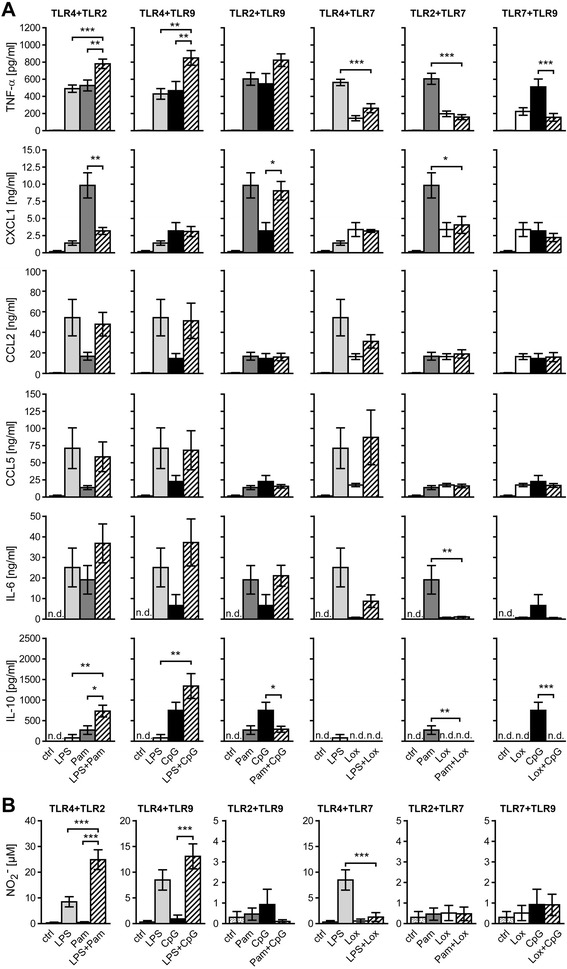
**Pairwise TLR activation results in a distinct profile of cytokines and chemokines in microglia.** Purified microglia were stimulated with LPS (100 ng/mL), Pam3CysSK4 (Pam, 100 ng/mL), loxoribine (lox, 1 mM), or CpG ODN (CpG, 1 μM) alone or simultaneously with pairwise combinations of the ligands, as indicated. PBS served as control. Supernatants were analyzed by (**A**) TNF-α ELISA (mean ± SEM of five independent experiments run with duplicates) and flow cytometry-based multiple analyte detection for several cytokine/chemokine levels, as indicated (mean ± SEM of four independent experiments) after 24 h, or by (**B**) Griess reaction for NO content (mean ± SEM of five independent experiments run with duplicates) after 48 h. ANOVA with Bonferroni-selected pairs of each individual ligand vs. combination of ligands, as indicated. P* <0.05; P** <0.005; P*** <0.001; n.d., not detected.

To characterize the inflammatory pattern induced by sole and pairwise TLR activation, the production of further cytokines and chemokines in microglia was determined by the bead-based assay FlowCytomix. Since the effect of simultaneous TLR stimulation on microglial activation in terms of TNF-α secretion was most prominent at 24 h, as described above, this incubation time was chosen in this experimental set-up (Figure 2A). Except for the anti-inflammatory cytokine IL-10, which was produced in response to TLR2, TLR4, and TLR9 activation, but not detected after activation of TLR7, individual stimulation of TLR2, TLR4, TLR7, and TLR9 induced a similar pattern of cytokines and chemokines including TNF-α, CXCL1, CCL2, IL-6, and CCL5, although to a different extent (Figure 2A). In contrast, IL-4, IL-17, IFN-γ, IL-12p70, IL-13, IL-1α, GM-CSF, and IL-23 were not detected in response to activation of any TLR tested during the time of observation (data not shown). Simultaneous stimulation of TLR4 plus TLR2 as well as TLR4 plus TLR9 led to additive levels of IL-6 and significant additive levels of IL-10 compared to activation of the respective single TLR. Activation of both TLR2 plus TLR4 and TLR2 plus TLR7 suppressed the production of CXCL1 compared to activation of TLR2 alone, whereas simultaneous activation of TLR2 plus TLR9 led to increased CXCL1 levels compared to sole activation of TLR9. In contrast, none of the pairwise ligand combinations led to significant modified expression levels of CCL2 or CCL5 compared to application of the respective single ligand. In general, co-stimulation of TLR7 tended to suppress the release of TNF-α, IL-6, CXCL1, and IL-10 induced by TLR4, TLR2, and TLR9 activation alone (Figure 2A). In supernatants of cells stimulated with agonists for TLR2 and TLR9, protein expression levels of all six analytes equaled the levels resulted from sole stimulation with Pam3CysSK4, suggesting that TLR2 signaling overruled that of TLR9 (Figure 2A).

TLR signaling also results in transcription of the enzyme NO synthase. Microglia activated by LPS release NO, which reveals toxic effects on neurons [17,28]. To further unravel the effect of sole and pairwise TLR stimulation on microglia, the amount of NO was measured in the respective supernatants by Griess reaction (Figure 2B). Relevant amounts of NO were not detected within 48 h after both activation of TLR4 alone and simultaneous TLR stimulation with LPS (data not shown). After 48 h, co-stimulation of TLR7 diminished NO secretion caused by activation of TLR4 alone, and co-stimulation of TLR4 plus TLR2 or plus TLR9 led to additive NO release. Additional stimulation of TLR2 significantly increased the release of NO in response to TLR4 activation by more than 2.5-fold by 48 h (Figure 2B) and up to 3-fold by 72 h (data not shown). In comparison, the effect of combined activation of TLR9 plus TLR4 was less pronounced with merely 1.5-fold more NO by 48 h (Figure 2B) and 72 h (data not shown) compared to the respective single TLR stimulation. Notably, the results in terms of NO release after stimulation of a single TLR versus two TLRs matched those of TNF-α, IL-6, and IL-10 secretion, as described above.

Taken together, pairwise stimulation of TLRs expressed in microglia results, time-dependently, in a pattern of inflammatory molecules that differs from the one obtained from activation of a single TLR.

### Stimulation of TLR4 in microglia with host-derived and pathogen-associated ligands leads to a distinct inflammatory response

**Figure 3:**
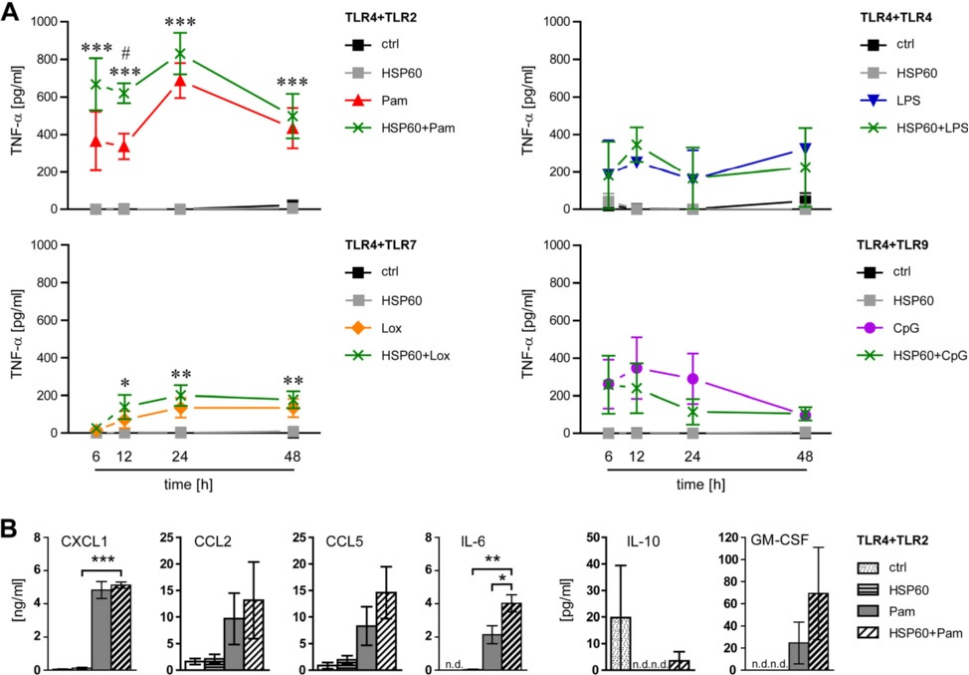
**Impact of TLR4 activation by HSP60 on the inflammatory response in microglia** ***in vitro*** **.** Primary microglia were stimulated with HSP60 (1 μg/mL), LPS (100 ng/mL), Pam3CysSK4 (Pam, 100 ng/mL), loxoribine (lox, 1 mM), or CpG ODN (CpG, 1 μM) alone or simultaneously with pairwise combinations of the ligands, as indicated. Supernatants were analyzed **(A)** by TNF-α ELISA at various time points, as indicated (mean ± SEM of two to four independent experiments run with duplicates) or **(B)** by flow cytometry-based multiple analyte detection for cytokine/chemokine levels, as indicated (mean ± SEM of three independent experiments run with duplicates) after 12 h. Data in **(A)** were analyzed by two-way ANOVA with Bonferroni-selected pairs of each individual compound vs. combination of compounds (*HSP60 vs. ligand combination; #Pam vs*.* ligand combination). P*, *#* <0.05; P** <0.005; P*** <0.001. Data in **(B)** were analyzed by ANOVA with Bonferroni-selected pairs of each individual compound vs. combination of compounds, as indicated. P* <0.05; P** <0.005; P*** <0.001; n.d., not detected.

The stress protein HSP60 released from injured CNS cells was identified as a host-derived ligand for TLR4 in microglia [18]. We wondered whether the outcome of TLR activation in microglia depends on the DAMP or pathogen-associated nature of the TLR ligands used. Thus, we repeated the experimental set-up using different pathogen-associated TLR ligands, as described above, and included the host-derived TLR4 ligand HSP60. In detail, microglia were stimulated with HSP60 alone or in combination with LPS (TLR4), Pam3CysSK4 (TLR2), loxoribine (TLR7), or CpG ODN (TLR9). Supernatants were analyzed for TNF-α content at several time points, as indicated (Figure 3A). Whereas stimulation of TLR4 in microglia with LPS resulted in the release of TNF-α during the whole round of observation (Figure 1), stimulation of TLR4 with HSP60 did not result in significant amounts of TNF-α secreted from microglia compared to control conditions at any time point. TNF-α levels remained unchanged in response to stimulation of TLR4 plus TLR9 with HSP60 and CpG ODN, respectively, and, surprisingly, of TLR4 with both HSP60 and LPS, compared with the respective single application of the PAMP. In contrast, release of TNF-α was increased in response to co-stimulation of TLR4 plus TLR7 with HSP60 and loxoribine compared to HSP60 alone. Combined activation of TLR4 plus TLR2 with HSP60 and Pam3CysSK4, respectively, resulted in an additive release of TNF-α compared to stimulation of TLR2 alone (Figure 3A). Maximum differences regarding secretion of TNF-α between cells treated with Pam3CysSK4 alone and cells incubated with the combination of Pam3CysSK4 and HSP60 were detected at 12 h (Figure 3A). Therefore, further analysis of the cytokine and chemokine profile after stimulation of TLR4 plus TLR2 with HSP60 and Pam3CysSK4, respectively, was performed at this time point (Figure 3B). Analysis of microglial supernatants by FlowCytomix revealed statistically significant differences between the combined stimulation with Pam3CysSK4 plus HSP60 and the respective single application regarding IL-6 secretion as well as between the combined stimulation and HSP60 alone regarding CXCL1 secretion. Furthermore, a trend towards increased secretion of CCL5 and GM-CSF after incubation with Pam3CysSK4 plus HSP60 compared to the respective single application was observed, although these results did not reach statistical significance (Figure 3B).

These findings indicate that activation of TLR4 by DAMPs and PAMPs leads to the induction of different intracellular signaling pathways in microglia.

### Co-stimulation of TLR4 and TLR2 in microglia causes enhanced neuronal injury compared to stimulation of the respective receptor alone

**Figure 4:**
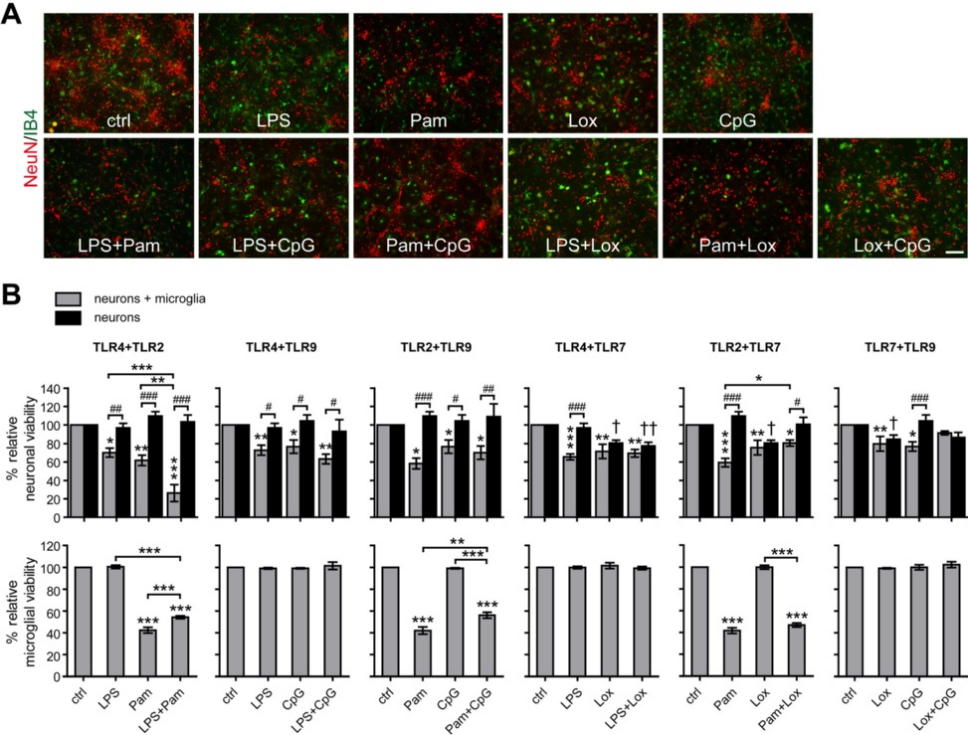
**Co-activation of TLR4 and TLR2 causes enhanced microglia-dependent neuronal injury** ***in vitro.*** **(A)** Primary cortical neurons were supplemented with purified microglia. Co-cultures were stimulated with 100 ng/mL LPS, 100 ng/mL Pam3CysSK4 (Pam), 1 mM loxoribine (lox), or 0.1 μM CpG ODN (CpG) alone or with pairwise combinations of the ligands, as indicated. PBS served as control. After 72 h co-cultures were immunostained with NeuN Ab (neurons, red) and IB4 (microglia, green). Scale bar, 100 μm. **(B)** Cortical neurons alone or supplemented with microglia were incubated with the TLR ligands named above, as indicated. After 72 h, NeuN^+^ and IB4^+^ cells were quantified and expressed as relative neuronal viability and relative microglial viability, respectively, as indicated. Each condition was performed in duplicate and averaged. Mean ± SEM from four to five individual experiments with ANOVA followed by Bonferroni post-hoc test of control vs*.* each treatment and of single vs. pair-wise stimulation (relative neuronal viability: co-cultures: *; neurons: †, relative microglial viability: co-cultures *), as indicated. Two-way ANOVA with Bonferroni post-hoc test between indicated groups testing if the neurotoxic effect is dependent on microglia (#). P* <0.05; P** <0.005; P*** <0.001; *P#* <0.05; *P##* <0.005; *P###* <0.001; *P*† <0.05; *P*†† <0.005.

Activation of TLR4 in microglia leads to neuronal cell death [20]. To investigate the impact of pairwise TLR activation on neuronal injury, co-cultures of neurons and microglia were exposed to the TLR ligands LPS (TLR4), Pam3CysSK4 (TLR2), loxoribine (TLR7), or CpG ODN (TLR9) alone, or to combinations of these ligands, as indicated (Figure 4A). Subsequent immunocytochemistry using NeuN antibody and IB4 revealed that all TLR ligands and ligand combinations tested resulted in loss of neurons compared to control conditions, although to a varying extent. To determine the neurotoxic effects’ dependency on microglia, purified neurons without microglia were stimulated in parallel (Figure 4B). Quantification of NeuN^+^ cells in co-cultures of neurons and microglia confirmed the neurotoxic effects described above. Comparative analysis of co-cultures and neurons alone revealed that LPS, Pam3CysSK4, and CpG ODN affected neuronal survival solely when microglia were present (Figure 4B), as expected [20,28,29]. In contrast, activation of TLR7 led to neuronal cell death in the absence of microglia (Figure 4B) due to cell-autonomous neurotoxic effects mediated by the same receptor [7]. Simultaneous stimulation of TLR4 plus TLR2 in co-cultures caused severe neurotoxicity reducing neuronal viability to 26.3% ± 20.6 compared to control conditions. These neurotoxic effects were significantly increased compared to the effects induced by TLR2 or TLR4 activation alone. Combined stimulation of TLR4 plus TLR9 showed merely a trend in reducing neuronal viability compared to the neuronal survival after stimulation of the respective single TLR. All other combinations of TLR ligands, as indicated, resulted in neuronal cell death to a similar extent as it was observed in cell cultures incubated with the respective ligand alone (Figure 4B). In addition, relative microglial viability in the respective co-cultures incubated with different TLR ligands and ligand combinations was assessed (Figure 4B). Treatment with the TLR2-specific ligand Pam3CysSK4 alone caused loss of microglia, as expected [30]. However, simultaneous stimulation of TLR2 plus TLR4 or TLR2 plus TLR9 led to a slight, but significant increase of microglial numbers compared to sole stimulation of TLR2. Treatment with CpG ODN, LPS, and loxoribine, alone or in combination, did not affect microglial viability compared to control conditions during the observed time course.

### Simultaneous activation of microglia by loxoribine, an agonist for TLR7, and the DAMP HSP60, an agonist for TLR4, causes enhanced neuronal injury compared to the respective single application

**Figure 5:**
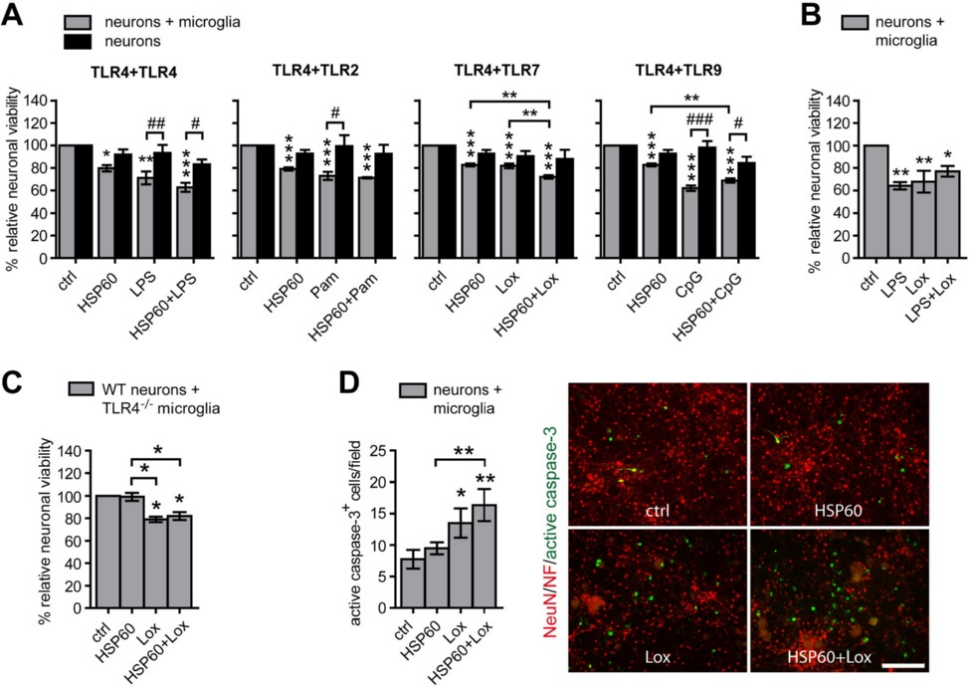
**Simultaneous activation of TLR4 by HSP60 and of TLR7 by loxoribine causes enhanced neuronal injury** ***.*** Co-cultures of neurons and microglia as well as neurons alone were stimulated with 1 μg/mL HSP60, 100 ng **(A)** or 1 μg **(B)** /mL LPS, 100 ng/mL Pam3CysSK4 (Pam), 1 mM loxoribine (lox), or 0.1 μM CpG ODN (CpG) alone or with pairwise combinations of the ligands, as indicated. PBS served as control. After 72 h, NeuN^+^ cells **(A** **-C)** or after 24 h, cells positive for activated caspase-3** (D)** were quantified and expressed as relative neuronal viability or caspase-3^+^ cells/field, respectively. Each condition was performed in duplicate and averaged. Mean ± SEM from **(A)** three to four and **(B,** **C)** four individual experiments with ANOVA followed by Bonferroni post-hoc test of control vs. each treatment and of single vs. pair-wise stimulation (*), as indicated. **(A)** In addition, data were analyzed by two-way ANOVA with Bonferroni post-hoc test between indicated groups testing if effect is dependent on microglia (#). **(D)** Mean ± SEM from four individual experiments with ANOVA followed by Bonferroni post-hoc test of control vs. each treatment and of single vs*.* pairwise stimulation. Scale bar, 100 μm. P* <0.05; P** <0.005; P*** <0.001; *P#* <0.05; *P##* <0.005; *P###* <0.001.

Next, we aimed at determining the impact of simultaneous TLR stimulation by pathogen-associated and host-derived ligands on the viability of neurons. To this end, co-cultures of neurons and microglia and neurons alone were stimulated with different DAMPs and pathogen-associated ligands and their combinations, as indicated, and were analyzed in terms of neuronal survival by immunocytochemistry (Figure 5A). Co-cultures of microglia and neurons incubated with the DAMP HSP60 plus the PAMP LPS, both activating TLR4, showed a slight trend of increased neuronal decay compared to the one induced by the respective single stimulus. Whereas combined stimulation of TLR4 plus TLR2 with LPS and Pam3CysSK4, respectively, resulted in increased neurotoxicity compared to the respective single application in co-cultures (Figure 4), neurotoxic effects were unchanged in co-cultures challenged with HSP60 plus Pam3CysSK4, thereby activating TLR4 and TLR2, compared with stimulation with the respective ligands alone, although the same TLR, namely TLR4, was involved (Figure 5A). Combination of the DAMP HSP60 plus the TLR7 agonist loxoribine led to a significant increase in neuronal cell death by a further 10% compared to the one induced by each ligand alone (Figure 5A). Simultaneous incubation of co-cultures derived from the same preparation analyzed above with high doses of LPS (TLR4) plus loxoribine (TLR7) did not result in increased neuronal cell death compared to the respective single challenge (Figure 5B). Thus, simultaneous activation of TLRs and subsequent neuronal injury may not only be dependent on the TLR *per se*, but also on the ligand engaged. Neurotoxic effects induced by HSP60 alone or in combination with the respective pathogen-associated TLR ligand required the presence of microglia regularly (Figure 5A). To confirm that the enhanced neurotoxic effects in co-cultures exposed to HSP60 plus loxoribine were dependent on TLR4 expressed in microglia, neurons were co-cultured with microglia derived from mice lacking TLR4 (Figure 5C). As expected, in these cell cultures, neurotoxicity induced by HSP60 alone was abolished. Moreover, co-cultures containing microglia deficient of TLR4 treated with the combination HSP60 plus loxoribine displayed similar neurotoxic effects as observed in cell cultures incubated with loxoribine alone, confirming the specificity of the respective TLR engaged. Immunostaining of co-cultures containing wild-type microglia with an antibody against activated caspase-3 showed that stimulation of TLR7 alone with loxoribine resulted in significantly enhanced apoptosis compared to control conditions. Combined stimulation with HSP60 plus loxoribine caused a significant increase in cells positive for activated caspase-3 compared with the single application of HSP60 (Figure 5D).

### Expression of different TLR mRNAs in response to activation of a single TLR in the brain

**Figure 6:**
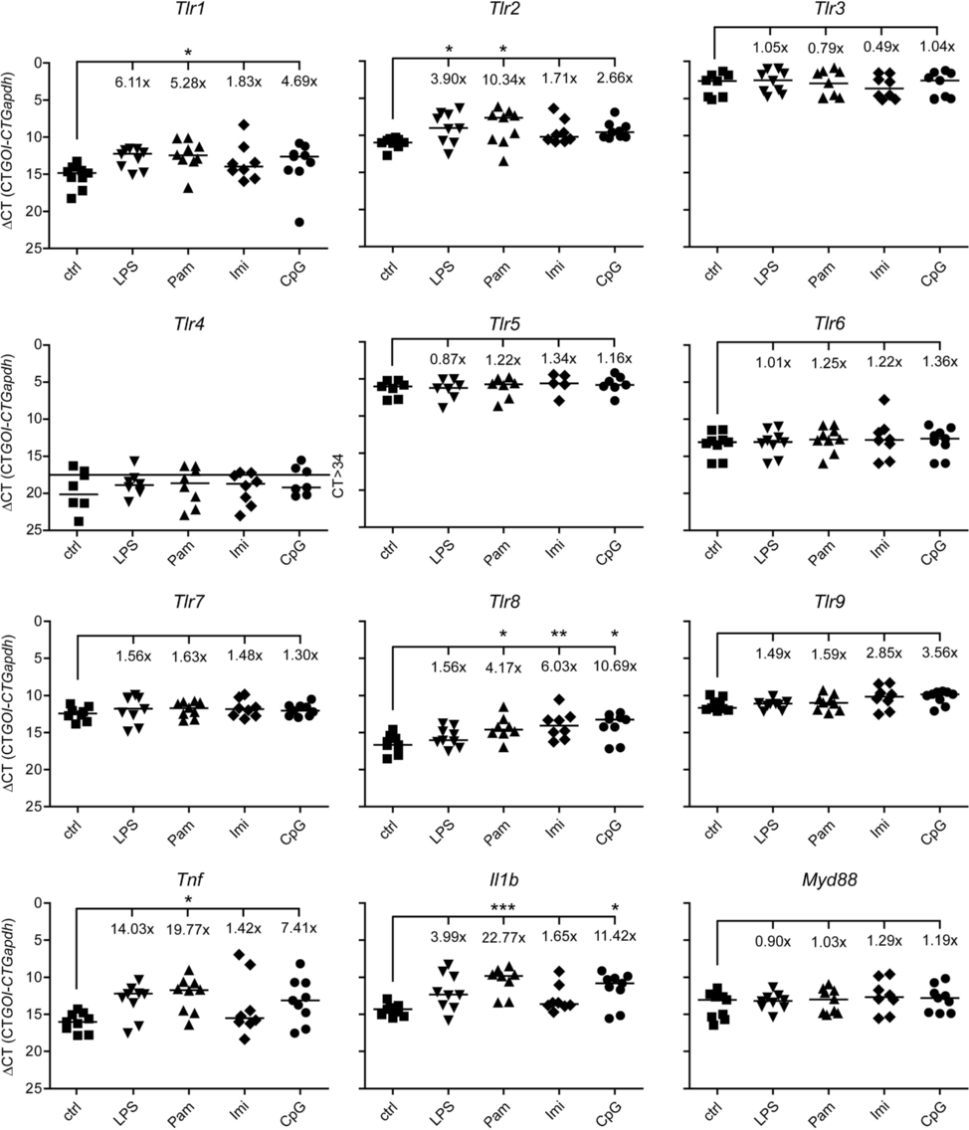
**mRNA expression of TLRs in response to activation of a single TLR in the CNS** ***.*** C57BL/6J mice were injected intrathecally with 10 μg LPS (n = 9), 10 μg Pam3CysSK4 (Pam, n = 9), 10 μg imiquimod (Imi, n = 8), or 10 μg CpG ODN (CpG, n = 9). Intrathecal injection of 0.9% NaCl (ctrl, n = 9) served as control. After 12 h, brain tissue was analyzed for mRNA expression of TLRs and proinflammatory molecules, as indicated, by quantitative real-time PCR. Data are presented as delta CT (dCT = CT_gene of interest_ – CT_*Gapdh*_) of each mouse with median per group on reverse scale to visualize changes in mRNA levels. Fold increase (2^-ddCT^, with fold change >2 and <0.5 expressing biological significance) was calculated with the median of each group, setting the control to 1; ANOVA of log2 transformed dCT values followed by Bonferroni post-hoc test of control vs. treatment. P* <0.05; P** <0.005; P*** <0.001.

Expression of TLR mRNA in both immune cells and cells of the CNS depends on the mode of infection, e.g., induced by bacteria, viruses, or parasites [12,31]. Several previous studies on infectious diseases indicate that multiple pattern recognition receptors act in concert to induce protective immune responses [32,33]. We performed a comprehensive study using intrathecal injection of different specific TLR ligands into mice [19] to determine the effect of the stimulation of a single TLR on the expression of various TLR family members, the adapter MyD88, and proinflammatory molecules, such as TNF-α and Il-1β, in the CNS compared to control conditions *in vivo*. In detail, brain tissue from mice injected intrathecally with LPS (TLR4), Pam3CysSK4 (TLR2), imiquimod (TLR7), or CpG ODN (TLR9) was analyzed by real-time PCR after 12 h (Figure 6). Whereas intrathecal injection of the TLR4 ligand LPS resulted in an increase in the expression especially of TLR1 and TLR2 mRNA as well as of *tnf* and *il1β*, injection of the TLR2 agonist Pam3CysSK4 led to an increase in the expression especially of TLR1, TLR2, TLR8 mRNA, *tnf*, and *il1β* compared to control conditions. Activation of TLR7 caused an increase in the expression especially of the endosome-related receptors TLR8 and TLR9 mRNA, and stimulation of TLR9 resulted in up-regulation especially of TLR1, TLR2, TLR8, TLR9 mRNA, *tnf*, and *il1β* compared to control conditions. Expression, especially of TLR3, TLR5, TLR6, TLR7, and MyD88 mRNA, was unaffected by stimulation of the respective TLR. In general, expression of TLR4 mRNA was nearly undetectable and did not change under any condition (Figure 6). Also, mRNA expression levels of the TLR downstream signaling molecules *irak1*, *irak4*, *traf6*, *nf-κb*, the cytokines *il4*, *il6*, *il10*, and *iNOS* were undetectable or very low under any condition and did not change in response to intrathecal stimulation of any TLR compared to control conditions (data not shown). Notably, activation of a TLR in the CNS by its specific ligand did not necessarily regulate the expression of the same TLR and therefore did not influence its own transcription (Figure 6). However, in our experimental set-up, statistical significance often did not reflect biological significance of a group (defined as <0.5 and >2-fold expression compared to control, respectively), due to large variations of CT values among the injected mice.

In summary, activation of a single TLR in the brain results in up-regulation of a distinct pattern of TLR family members and TLR-associated molecules *in vivo*.

### Sole activation of TLR2, TLR7, and TLR9 in the CNS leads to microglial activation and neuronal damage *in vivo*

**Figure 7:**
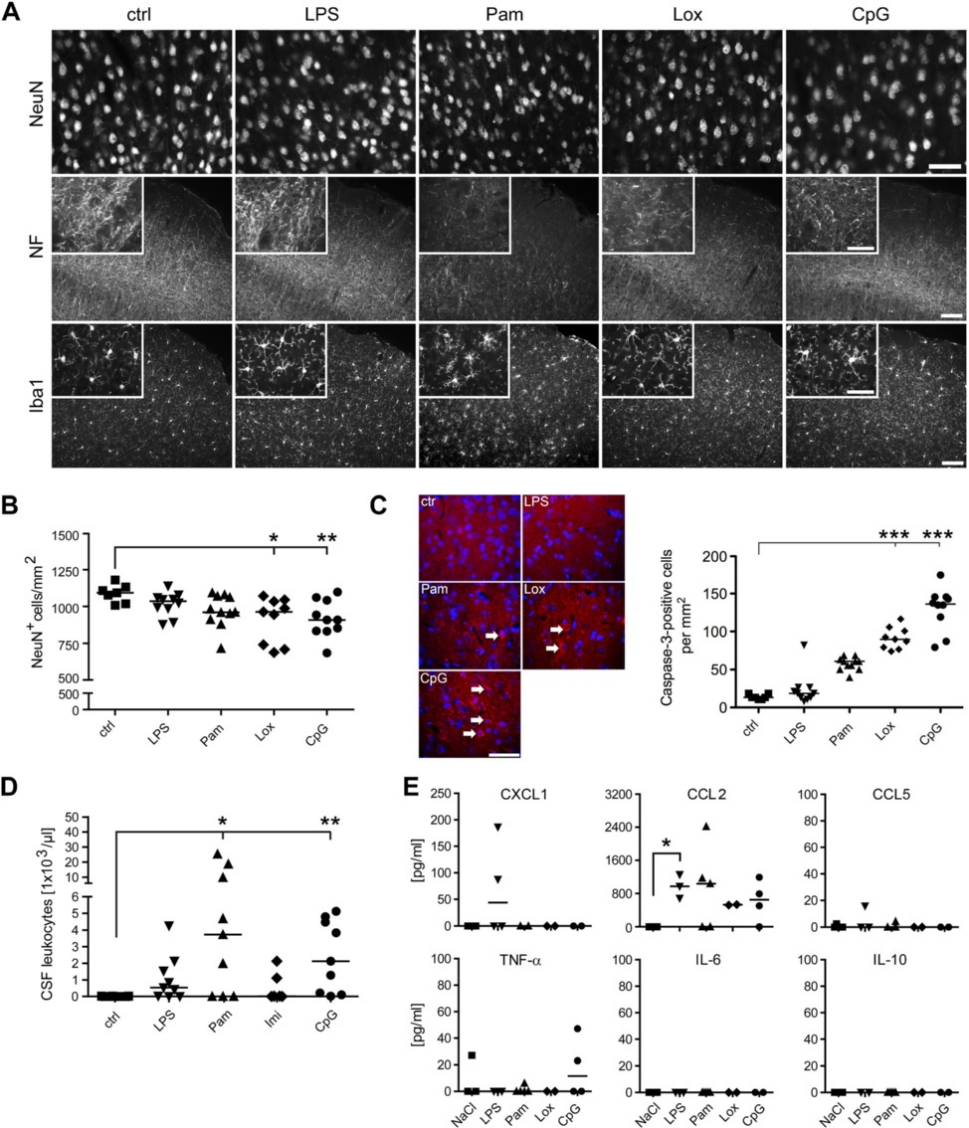
**Activation of specific TLRs causes neurodegeneration and inflammation in the brain** ***in vivo.*** **(A,** **B) **C57BL/6J mice were injected intrathecally with water (control, n = 7) or one of the four TLR ligands (10 μg LPS, n = 10; 40 μg Pam3CysSK4, Pam, n = 11; 136 μg loxoribine, Lox, n = 9; 10 μg CpG ODN, CpG, n = 10), as indicated. After 3 days, brains were analyzed by immunohistochemistry. **(A)** Representative micrographs of the cerebral cortex immunostained with NeuN Ab, neurofilament Ab, and Iba1 Ab to detect neurons, axons, and microglia, respectively. Scale bar, 100 μm, inset 50 μm. **(B)** NeuN^+^ cells of the cerebral cortex were quantified. The mean of six high power fields per cerebral cortex is expressed as NeuN^+^ cells per mm^2^; median with Kruskal-Wallis test followed by Bonferroni post-hoc test of control vs. treatment. P* <0.05; P** <0.005. **(C)** Representative micrographs of the cerebral cortex immunostained with an Ab against activated caspase-3. Cortical cells positive for activated caspase-3, as indicated by arrows, were quantified. The mean of six high power fields per cerebral cortex is expressed as caspase-3^+^ cells per mm^2^; median with Kruskal-Wallis test followed by Bonferroni post-hoc test of control vs. treatment. P*** <0.001. **(D)** C57BL/6J mice were injected intrathecally with 0.9% NaCl (control, n = 9) or one of the four TLR ligands (10 μg LPS, n = 9; 10 μg Pam3CysSK4, Pam, n = 9; 10 μg imiquimod, Imi, n = 8; 10 μg CpG ODN, CpG, n = 9), as indicated. After 12 h, cerebrospinal fluid (CSF) was obtained from the cistern magna. Leukocytes in the CSF were counted and are expressed as leukocytes per μL CSF. Median with Kruskal-Wallis test followed by Dunn’s multiple comparison test of control vs. treatment. P* <0.05; P** <0.005. **(E)** C57BL/6J mice were injected intrathecally with 0.9% NaCl (control, n = 4) or one of the four TLR ligands (10 μg LPS, n = 4; 10 μg Pam3CysSK4, Pam, n = 4; 10 μg loxoribine, Lox, n = 4; 10 μg CpG ODN, CpG, n = 4), as indicated. After 24 h, CSF was analyzed by flow cytometry-based multiple analyte detection for amounts of various cytokines/chemokines, as indicated. Median with Kruskal-Wallis test followed by Dunn’s multiple comparison test of control vs*.* treatment. P* <0.05.

We have utilized intrathecal injection of agonists highly specific for the respective TLR to establish an experimental model in the mouse that accurately reflects neuronal damage and neuroinflammation, providing an excellent model system to investigate effects on the brain mediated by activation of a single TLR [7,19]. Whereas TLR2 and TLR4 mediate neuronal damage through activation of microglia, activation of TLRs expressed in neurons, such as TLR7, can cause cell-autonomous neuronal injury *in vitro* [16]. To assess the impact of different activated TLRs on inflammation and neurodegeneration in the brain *in vivo*, the TLR ligands LPS (TLR4), Pam3CysSK4 (TLR2), loxoribine (TLR7), or CpG ODN (TLR9) were injected intrathecally into mice. After 3 days, brain sections were immunostained with markers for neurons, axons, and microglia to analyze neuronal survival and microglial activation. Seven out of 9 mice injected with loxoribine and 7 out of 10 mice injected with CpG ODN revealed loss of neurons and axonal injury in the cerebral cortex compared to control conditions, respectively (Figure 7A). In both groups, especially in areas with reduced neurofilament-positive fibers, activated microglia displayed a pronounced immunoreactivity for Iba1 and thicker processes compared to control conditions (Figure 7A). Quantification of NeuN^+^ cells revealed that upon stimulation of TLR7 or TLR9 neuronal numbers in the cerebral cortex were reduced by 11.9% and 17%, respectively (Figure 7B). Activation of TLR2 also resulted in loss of neurons by 12.1%, although not reaching statistical significance, and caused major injury of axons in 9 out of 11 mice (Figure 7A,B). Furthermore, microglia of brains challenged by the TLR2 agonist displayed a distinct activated state (Figure 7A). Similarly, activated microglia in the cerebral cortex were observed in response to intrathecal LPS. However, stimulation of TLR4 did not lead to a significant decrease of neuronal numbers or major axonal injury compared to control conditions (Figure 7A,B). Immunostaining of brain sections with an antibody against activated caspase-3 confirmed the induction of apoptosis in the cerebral cortex of animals injected with loxoribine and CpG ODN (Figure 7C).

Infiltration of peripheral macrophages and leukocytes into the brain is restricted until they are attracted by signals generated during infection or injury [34]. To assess the inflammatory response in the periphery after activation of TLRs in the CNS *in vivo*, leukocytes in the CSF of mice injected intrathecally with different TLR ligands were quantified (Figure 7D). Whereas leukocytes were absent in the CSF of control mice, intrathecal injection of Pam3CysSK4 (TLR2), LPS (TLR4), imiquimod (TLR7), and CpG ODN (TLR9) led to infiltration of leukocytes. By comparison, the strongest influx was detected after injection of Pam3CysSK4 and CpG ODN. In contrast, intrathecal injection of imiquimod (Figure 7D) and loxoribine (data not shown), both activating TLR7, or LPS, provoked the recruitment of only minute numbers of leukocytes into the CSF. CSF probes of mice injected intrathecally with the TLR ligands named above were also analyzed by FlowCytomix regarding the presence of various cytokines and chemokines, as indicated, after 24 h (Figure 7E). Whereas injection of the respective TLR agonist resulted in undetectable or only minute amounts of CXCL1, CCL5, and TNF-α, CCL2 secretion after injection of LPS was significantly increased compared to control conditions. IL-6 and IL-10 were not detected under any condition.

In summary, activation of different TLRs in the CNS leads to microglial activation, influx of peripheral immune cells, secretion of distinct inflammatory mediators, and neurodegeneration in varying degrees depending on the specific TLR engaged *in vivo.*

### Subsequent challenge with the DAMP HSP60, an agonist for TLR4, does not modify neurodegeneration induced by TLR2 or TLR7 in the CNS

**Figure 8:**
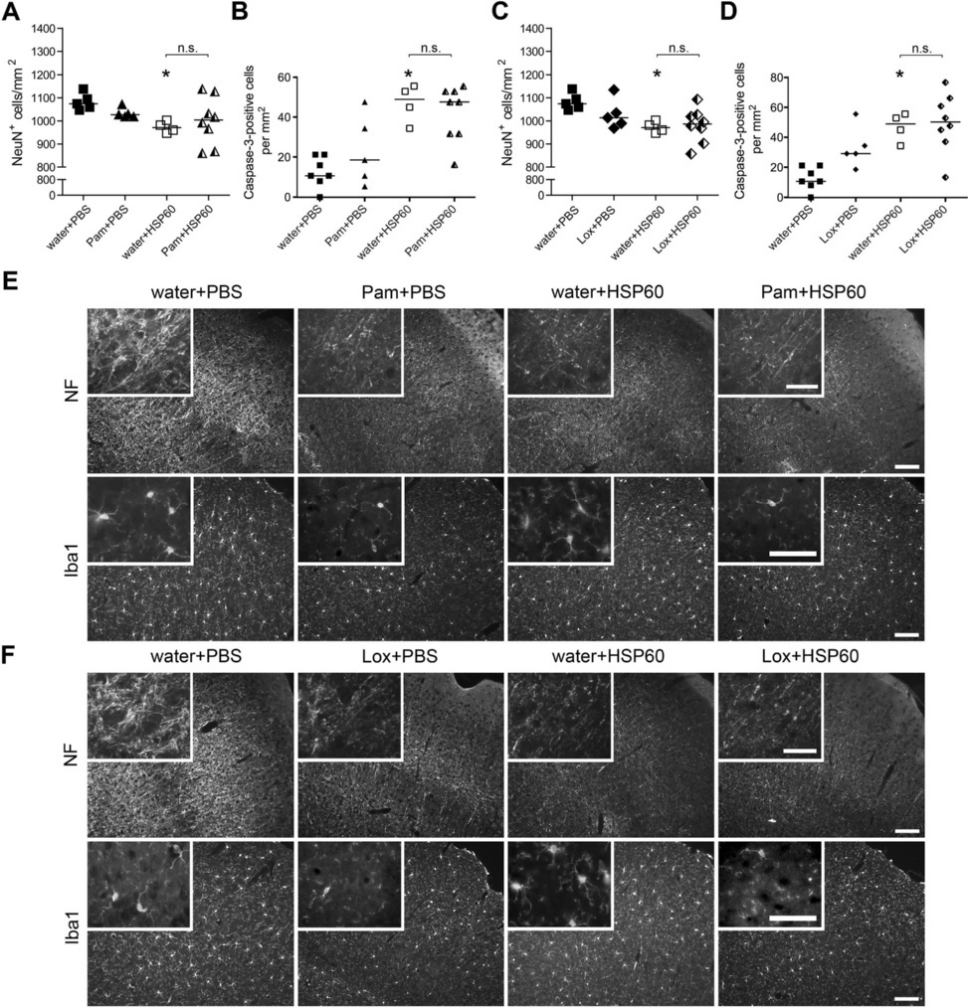
**Subsequent challenge with HSP60 does not exacerbate CNS damage induced by exogenous TLR ligands.** C57BL/6J mice received two subsequent intrathecal injections. On day one 40 μL water (carrier), 40 μg Pam3CysSK4 (Pam), or 136 μg loxoribine (Lox) were injected. After 24 h, 40 μL PBS or 40 μg HSP60 were injected additionally (water + PBS n = 7; water + HSP60 n = 4; Pam + PBS n = 5; Lox + PBS n = 5; Pam + HSP60 n = 8; Lox + HSP60 n = 8). After a further 3 days, brains were analyzed by immunohistochemistry. **(A, C)** NeuN^+^ cells of the cerebral cortex were quantified, and the mean is expressed as NeuN^+^ cells per mm^2^; median with Kruskal-Wallis test followed by Bonferroni post-hoc test of control (water + PBS) vs. treatment or between the respective treatments, as indicated. P* <0.05; n.s.: not significant. **(B,** **D)** Brain sections of injected mice were immunostained with an Ab against activated caspase-3. Cortical cells positive for activated caspase-3 were quantified. The mean of six high power fields per cerebral cortex is expressed as caspase-3^+^ cells per mm^2^; median with Kruskal-Wallis test followed by Bonferroni post-hoc test of control (water + PBS) vs. treatment or between the respective treatments, as indicated. P* <0.05; n.s.: not significant. **(E,** **F)** Representative micrographs of the cerebral cortex immunostained with neurofilament Ab (NF) and Iba1 Ab to mark axons and microglia, respectively. Scale bar, 100 μm, inset 50 μm.

Next, we addressed the question of whether stimulation of TLRs by DAMPs, which are released from injured cells [35], adds to previous or ongoing TLR-induced CNS injury *in vivo*. We hypothesized that additional activation of TLRs by DAMPs fuels the response of the innate immune system induced by PAMPs, thereby exacerbating tissue damage. In detail, the PAMPs Pam3CysSK4 (Figure 8A,B) or loxoribine (Figure 8C,D) were injected intrathecally into mice, thereby stimulating TLR2 and TLR7, respectively. Endotoxin-free water, in which Pam3CysSK4 and loxoribine were dissolved, was injected as a carrier control. After 24 h, animals received an intrathecal injection of HSP60. PBS, in which HSP60 was dissolved, served as additional carrier control. After a further 3 days, brains were subjected to immunohistochemical analysis. Quantification of NeuN-positive cells in the cerebral cortex revealed a significant loss of 9.3% after injection of HSP60 (+water) and a tendential loss of 4.4% and 5.7% in response to injection of Pam3CysSK4 (+PBS) and loxoribine (+PBS, *P* = 0.0652, Mann-Whitney U test), respectively, compared to control conditions (water + PBS) (Figure 8A,C). Brains of mice that received additional HSP60 after stimulation of TLR2 (Pam + HSP60) or TLR7 (Lox + HSP60) displayed no significant changes regarding neuronal survival in the cerebral cortex compared to brains challenged by the respective single ligand (Figure 8A,C). Immunostaining of brain sections with an antibody against activated caspase-3 confirmed the induction of apoptosis in animals injected with HSP60 (+water) and the absence of additional degenerating effects compared to treatment with the respective single ligand if the ligand combinations named above were used (Figure 8B,D). Also, we tested for the integrity of axons by using an antibody against neurofilaments (Figure 8E,F). In accordance with the results regarding neuronal viability in the cerebral cortex described above, axonal injury was not enhanced by the additional injection of HSP60 compared to the respective single injection (Figure 8E,F).

Brains of animals treated with HSP60 (+water) showed similar numbers and morphologic characteristics of microglia compared with control conditions (Figure 8E,F). In brains challenged by TLR2 stimulation (Pam + PBS) less microglia compared to control conditions were detected, and the morphology of these cells resembled a resting state (Figure 8E). However, in this experiment, mice were analyzed 24 h later than in the previous experiment in which, on the contrary, distinct activation of microglia was observed (Figure 7). Reduction in microglial numbers after an extended time period might be a consequence of TLR2-mediated microglial death [36]. Similar morphologic characteristics of microglia were observed in brains activated by TLR2 (Pam3CysSK4) plus TLR4 (HSP60) (Figure 8E). In animals injected with the TLR7 agonist loxoribine (Lox + PBS) or with a combination of loxoribine plus the TLR4 agonist HSP60 microglia likewise displayed a resting state, and numbers of these cells were decreased compared with control conditions and animals treated with HSP60 (+water) (Figure 8F).

Taken together, sole activation of TLR2 by PamCysSK4, TLR7 by loxoribine, and TLR4 by its host-derived ligand HSP60 cause neurodegeneration, whereas activation of TLR4 by its pathogen-associated ligand LPS does not significantly affect neuronal viability *in vivo*. Although doses of the respective ligands and in particular timing, which we did not test, have to be taken in account, these results demonstrate that CNS inflammation and injury induced by TLR2 or TLR7 activation is not exacerbated by subsequent activation of TLR4 through the PAMP HSP60.

## Discussion

Activation of TLRs contributes to both infectious and non-infectious CNS diseases [13]. So far, common approaches to unravel the influence of TLRs in the CNS used mice deficient of one TLR or of their signaling adaptors or made use of a single ligand that is highly specific for activation of the respective TLR. However, although the pathophysiological relevance of the different TLR ligands for the brain has not yet been conclusively clarified, it can be reasonably assumed that more than one TLR is involved in physiological and pathological processes within the CNS, and molecules that activate TLRs are likely be present as a mixture at one time. We sought to analyze the impact of single and pairwise TLR activation on inflammation and neurodegeneration in the CNS.

Studies on models of systemic infectious diseases indicate that TLR family members act in concert to induce an effective antibacterial response [32,33]. This concept of receptor redundancy certainly makes sense, especially in the CNS, since pathogens are capable of eliciting devastating consequences in a tissue that has only limited regenerative capacity, such as the brain. Therefore, the host repertoire of available TLRs should be substantial, ensuring that an effective immune response will be rapidly induced upon infection of the CNS parenchyma. For example, although TLR2 is established as the major receptor for gram-positive bacteria, several studies on the brain indicate that TLR2 might be dispensable in the recognition of whole gram-positive bacteria [37]. Further, TLR signaling in the brain must be tightly controlled in order to respond properly to pathogens. Insufficient TLR signaling may result in susceptibility of infection, whereas excessive signaling may lead to septic shock or autoimmune diseases. Although TLR2, TLR4, TLR7, and TLR9 in microglia were shown to activate the canonical pathway resulting in the production of proinflammatory molecules [16,22], not all TLRs tested acted in synergy in our present study. While combined stimulation of TLR4 plus TLR2 and TLR4 plus TLR9 caused additive secretion of several inflammatory molecules, including TNF-α, IL-6, IL-10, CXCL1, and NO from microglia compared to activation of the respective single TLR, combined stimulation with ligands specific for TLR2 and TLR9 resulted in additive secretion of TNF-α, but did not induce such an enhanced response for the other tested inflammatory molecules.

The synergy of TLR4 and TLR2 activation on TNF-α secretion has been previously observed in macrophages [38], and it was postulated that confirmation of one pathogen-associated molecule through another might serve as a mechanism of safety for the organism [39]. Although not determined yet, it is possible that, in combination, specific TLR ligands activate both the signaling pathway through MyD88 and the one through TRIF at the same time [40]. This would explain why pairwise stimulation of TLR4 (via MyD88/TRIF) plus TLR9 (via MyD88), but not TLR2 (via MyD88) and TLR9 (via MyD88), resulted in the enhanced secretion of multiple inflammatory molecules from microglia in our experimental set-up. Further, the synergistic production of TNF-α might be, at least in part, due to a prolonged half-life of TNF-α mRNA [41], whereas the increase in NO might be due to an enhanced iNOS mRNA expression [42]. However, autocrine and/or paracrine features of the respective cytokine/chemokine may also contribute to an enhanced inflammatory response [43]. The fact that additional activation of TLR7 suppressed the inflammatory response induced by TLR2, TLR4, and TLR9 in microglia may result from interactions that have been described before in both peripheral immune cells and various cell lines. For example, inhibition of TLR7 by TLR9 through physical interaction was observed in HEK293 cells [44]. Ligands of TLR7 inhibit TLR9-induced IFN-α secretion from plasmacytoid dendritic cells and B cells, which was found unlikely due to kinetic up-take advantages [45] and was rather accounted to a subsequent reduced expression of the transcriptional factor IRF-7 [46]. Another study showed that both TLR7 and its agonist imiquimod, independent of TLR7, can exert inhibitory effects on TLR9-induced cytokine responses in glial cells [47].

While TLR7 and TLR9 primarily share the same compartment, namely the endosome, and TLR7 can exert a physical effect on TLR9, TLR2, and TLR4, whose activation is impaired by simultaneous activation of TLR7 in microglia, both TLR2 and TLR4 are primarily sited at the cell surface. It is possible, since TLR2 and TLR4 require recruitment of the adaptor Mal in addition to MyD88, that TLR7 signaling is initiated faster and suppresses consecutive signaling induced by TLR2 and TLR4 activation. Still, TLR2 and TLR4 primarily located on the cell membrane are readily available, while activation of the endosomal TLR7 requires internalization of its ligand. On the other hand, TLRs may compete for the same signaling molecules and the molecular complex assembled for TLR7 signaling may require fewer components than the one for TLR2 and TLR4, making it faster and more “favorable”.

Overall, we cannot rule out that specific cytokines, chemokines, and/or neurotoxic metabolites different from the ones investigated in our study accumulate in response to activation of the respective TLR or that the period of observation in our set-up was too short. Additionally, expression of distinct inflammatory molecules in the brain may depend on the concentrations of the different TLR ligands, which we did not test entirely. Finally, it has to be taken into account that microglia easily and rapidly change from a resting to an activated status, since they are extremely sensitive to virtually any change in their environment [48]. As a consequence, the extent of inflammatory responses may vary slightly between different experiments, depending on the activation status of the respective cell preparation.

Neuronal injury mediated via activation of TLR2 [28], TLR4 [20], and TLR9 [29] expressed in microglia has been described *in vitro*. In addition, activation of TLR7 in neurons results in cell-autonomous and microglia-mediated neurodegeneration [7]. Proinflammatory mediators, such as TNF-α [29] and NO [18], reveal neurotoxic effects in the context of TLR-induced neuronal injury via activation of microglia. However, to our knowledge, the impact of combined TLR stimulation on neuronal survival in the CNS has not been systematically studied so far. The impact of specific pairwise TLR stimulation in microglia on the inflammatory response and subsequent neuronal injury compared to activation of a single TLR is clearly reflected by the results from our present studies. For example, specific activation of both TLR4 and TLR2 revealed a synergistic effect on enhancing the inflammatory response and on reducing neuronal viability. However, this effect was only seen if the PAMP LPS was used for TLR4 activation. The fact that the host-derived TLR4 ligand HSP60 did not enhance the inflammatory response and/or neuronal injury induced by simultaneous TLR2 activation point to different intracellular signaling pathways triggered by LPS and HSP60 via the same TLR. However, these results indicate that, in principle, simultaneous activation of specific TLRs in the CNS can exert a detrimental effect on neuronal survival through an exaggerated inflammatory response.

In line with the concept of an exaggerated innate immune response in pathological states of the CNS, permanent intracerebral infusion of CpG ODN leads to pronounced microglial activation and axonal injury dependent on TLR9 [49]. Additionally, systemic stimulation of TLR4 with LPS prior to a sub-clinical hypoxic-ischemic insult causes severe loss of axons and neurons in mice [20]. Furthermore, stroked mice that received an intracerebral injection of high-mobility group box-1, another host-derived TLR4 ligand besides HSP60, display an increased edema, infarct area, and enhanced neurologic deficits compared to animals without this additional injection [50]. In the latter cases, mice deficient of TLR4 are protected against the respective CNS injury, indicating that additional TLR4 activation to the initial ischemic insult is detrimental. In our study, we set up an experiment in which the murine brain was challenged by the DAMP HSP60, a TLR4 agonist, after initial treatment with the respective pathogen-associated ligands for TLR2 or TLR7. Notably, we have demonstrated in previous work that neurotoxic effects mediated by HSP60 are not caused by contamination with LPS [18]. In the current study, mice displayed considerable loss of neurons and/or injury of axons in the brain if challenged by intrathecal HSP60 or the respective ligands for TLR2 and TLR7 alone. However, additional injection of HSP60 failed to deteriorate initial CNS injury induced by TLR2 or TLR7 activation. It is possible that these findings reflect the tolerance paradigm observed in several studies, in which immune cells stimulated with a TLR ligand react hyporesponsively to subsequent TLR activation [38,51]. A similar phenomenon termed pre-conditioning is frequently observed in the ischemic CNS. In this context, the murine brain is resistant to an ischemic insult, which occurs subsequently to a previous mild ischemic insult. This effect can be induced by prior challenge with LPS or other forms of sub-clinical stress. In the context of an experimental pre-conditioning event, systemic application of LPS, Pam3CysSK4, or CpG ODN, activating TLR4, TLR2, and TLR9, respectively, prior to an experimental stroke results in protection of the mouse brain [52-55]. Accordingly, TNF-α released after the primary stimulus was found to exert a regulatory effect on neuroprotection [53,54]. Mice lacking TLR4 receiving pre-conditioning prior to permanent cerebral ischemia are not protected and show reduced NF-κB activation and lower expression levels of TNF-α compared to wild-type animals [56].

Overall, these studies point to a complex modulation of inflammatory processes, protecting the brain from further damage mediated by inflammation to come. A variety of inhibitory strategies in TLR signaling in immune cells causing tolerance to subsequent stimulation have been elucidated. For example, signaling molecules are degraded or dissociation from another is decreased whereas expression of inhibitory proteins is enhanced [57]. Our finding that intrathecal HSP60 does not induce morphologic characteristics suggestive of activation of microglia *in vivo* does not exclude a contribution of microglia to neuronal injury, as was reported in our previous *in vitro* studies [18]. Subtle activation of glial cells *in vivo* likely occurs, as these cells constantly monitor their microenvironment [58], even though their gross morphology is unchanged. Furthermore, inflammatory responses induced by HSP60 through TLR4 and TLR2 in peripheral immune cells are dependent on the respective pathological context *in vivo* [59], a finding that is likely to apply to microglia as well. The finding that intrathecal LPS does not lead to significant loss of neurons in the cerebral cortex while such neurodegenerative effects occur in co-cultures of microglia and neurons [20] suggests that the situation in the brain *in vivo* is likely a lot more complex.

## Conclusions

Based on our results we conclude that the outcome of neuroinflammation and neuronal injury induced by an activated innate immune system in the CNS depends, at least in part, on the identity and combination of the TLR family members engaged. While activation of each of the tested TLRs provoked an inflammatory response in microglia and stimulation of selected TLRs caused neuronal damage, the extent and pattern of such a response was modified specifically by the TLR ligand used, the number and combination of activated TLRs, and the timing. Although all tested TLRs share common signaling pathways, not all of them synergized but also antagonized one another when activated simultaneously. A detailed understanding of the innate immunity’s action and mechanisms of fine-tuning the inflammatory response involved in both CNS homeostasis and disorders is essential and may pave the way for new preventive and therapeutic strategies.

## Additional files

Additional file 1: Figure S1. Secretion of TNF-α from microglia and viability of neurons in response to different TLR ligands. **(A)** Purified microglia from C57BL/6J mice were incubated for 6 h with various doses of LPS, Pam3CysSK4 (Pam), loxoribine (lox), CpG ODN (CpG), or HSP60, as indicated. PBS served as control. Supernatants were analyzed by TNF-α ELISA. Results are presented as mean ± SEM of three independent experiments run with duplicates. n.d., not detected. **(B)** Cortical neurons from C57BL/6J mice were incubated for 6 h with various doses of LPS, Pam3CysSK4 (Pam), loxoribine (lox), CpG ODN (CpG), or HSP60, as indicated. PBS served as control. Subsequently, cultures were immunostained with NeuN Ab to mark neurons, and NeuN-positive cells per field were quantified. *P** < 0.05, *P*** < 0.005, ANOVA with Bonferroni-selected two pairs of each dose of the respective ligand vs. control, as indicated. Results are presented as mean ± SEM of three independent experiments run with duplicates. Additional file 2: Figure S2. The TLR3-specific ligand poly(I:C) does not suppress TNF-α secretion from microglia incubated with other TLR ligands. **(A)** Purified microglia from C57BL/6J mice were incubated for 6 h with various doses of poly(I:C), as indicated. PBS served as control. Supernatants were analyzed by TNF-α ELISA. Results are presented as mean ± SEM of three independent experiments run with duplicates. n.d., not detected. **(B)** Purified microglia from C57BL/6J mice were incubated for 6 h with LPS (100 ng/mL), Pam3CysSK4 (Pam, 100 ng/mL), CpG ODN (CpG, 1 μM), or poly(I:C) (1 μg/mL) alone or simultaneously with pairwise combinations of the ligands, as indicated. PBS served as control. Supernatants were analyzed by TNF-α ELISA. Results are presented as mean ± SEM of three independent experiments run with duplicates. ANOVA with two Bonferroni-selected pairs of each individual ligand vs. ligand combination, as indicated. *P*** < 0.005, *P**** < 0.001, n.s., not significant.

## Abbreviations

CNS: Central nervous system
CpG ODN: cytosine-phosphate-guanine motifs
CSF: cerebrospinal fluid
DAMPs: damage-associated molecular patterns
GFAP: anti-glial fibrillary acidic protein
HSP60: Heat shock protein 60
LPS: Lipopolysaccharide
MyD88: myeloid differentiation primary response gene 88
NO: nitric oxide
PAMPs: pathogen-associated molecular patterns
PBS: phosphate buffered saline
PFA: paraformaldehyde
TLRs: Toll-like receptors
TLR4KO: TLR4 knock out
TRIF: TIR-domain-containing adaptor inducing IFN-β
