# Supplementary material for: The impact of single and pairwise Toll-like receptor activation on neuroinflammation and neurodegeneration
Source: J Neuroinflammation. 2014 Sep 20;11:166. doi: 10.1186/s12974-014-0166-7 (PMC4182775; doi:10.1186/s12974-014-0166-7)
Supplement: Additional file 1: Figure S1. — Secretion of TNF-α from microglia and viability of neurons in response to different TLR ligands. (A) Purified microglia from C57BL/6J mice were incubated for 6 h with various doses of LPS, Pam3CysSK4 (Pam), loxoribine (lox), CpG ODN (CpG), or HSP60, as indicated. PBS served as control. Supernatants were analyzed by TNF-α ELISA. Results are presented as mean ± SEM of three independent experiments run with duplicates. n.d., not detected. (B) Cortical neurons from C57BL/6J mice were incubated for 6 h with various doses of LPS, Pam3CysSK4 (Pam), loxoribine (lox), CpG ODN (CpG), or HSP60, as indicated. PBS served as control. Subsequently, cultures were immunostained with NeuN Ab to mark neurons, and NeuN-positive cells per field were quantified. P* < 0.05, P** < 0.005, ANOVA with Bonferroni-selected two pairs of each dose of the respective ligand vs. control, as indicated. Results are presented as mean ± SEM of three independent experiments run with duplicates. [file 12974_2014_166_MOESM1_ESM.pdf]

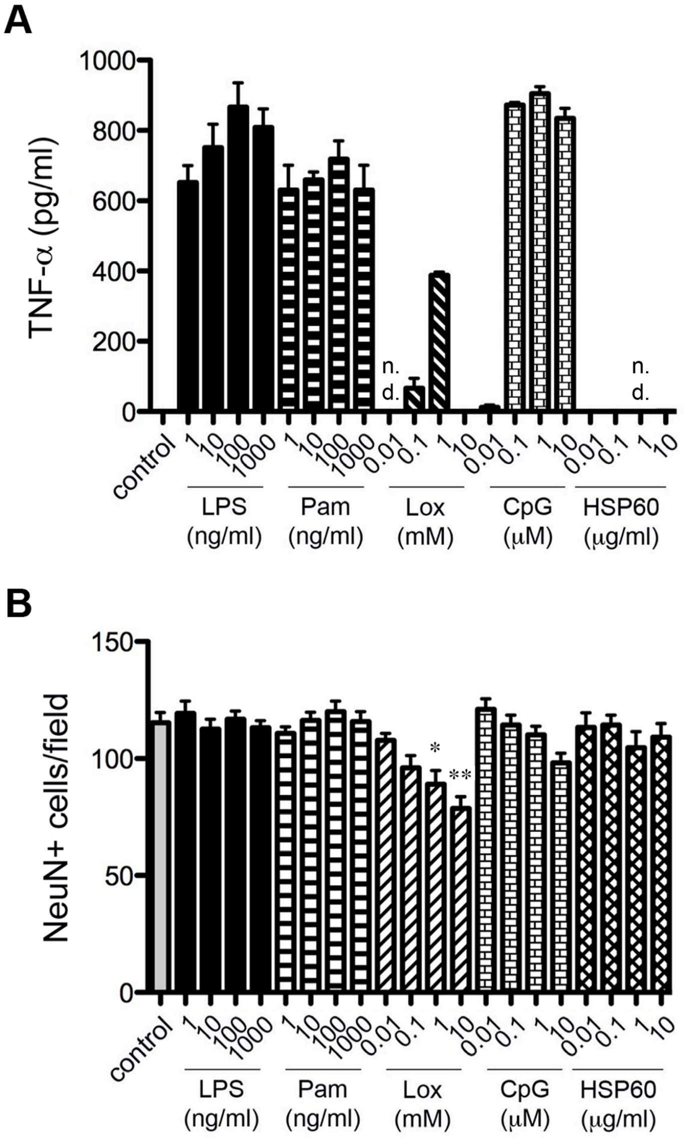

Supplementary Figure 1. Secretion of TNF-α from microglia and viability of neurons in response to different TLR ligands. (**A**) Purified microglia from C57BL/6J mice were incubated for 6 h with various doses of LPS, Pam3CysSK4 (Pam), loxoribine (lox), CpG ODN (CpG), or HSP60, as indicated. PBS served as control. Supernatants were analyzed by TNF-α ELISA. Results are presented as mean ± SEM of 3 independent experiments run with duplicates. n.d., not detected. (**B**) Cortical neurons from C57BL/6J mice were incubated for 6 h with various doses of LPS, Pam3CysSK4 (Pam), loxoribine (lox), CpG ODN (CpG), or HSP60, as indicated. PBS served as control. Subsequently, cultures were immunostained with NeuN Ab to mark neurons, and NeuN-positive cells per field were quantified. $p^{*}<0.05$, $p^{**}<0.005$, ANOVA with Bonferroni-selected

pairs of each dose of the respective ligand *vs.* control, as indicated. Results are presented as mean ± SEM of 3 independent experiments run with duplicates.
