# Supplementary material for: The impact of single and pairwise Toll-like receptor activation on neuroinflammation and neurodegeneration
Source: J Neuroinflammation. 2014 Sep 20;11:166. doi: 10.1186/s12974-014-0166-7 (PMC4182775; doi:10.1186/s12974-014-0166-7)
Supplement: Additional file 2: Figure S2. — The TLR3-specific ligand poly(I:C) does not suppress TNF-α secretion from microglia incubated with other TLR ligands. (A) Purified microglia from C57BL/6J mice were incubated for 6 h with various doses of poly(I:C), as indicated. PBS served as control. Supernatants were analyzed by TNF-α ELISA. Results are presented as mean ± SEM of three independent experiments run with duplicates. n.d., not detected. (B) Purified microglia from C57BL/6J mice were incubated for 6 h with LPS (100 ng/mL), Pam3CysSK4 (Pam, 100 ng/mL), CpG ODN (CpG, 1 μM), or poly(I:C) (1 μg/mL) alone or simultaneously with pairwise combinations of the ligands, as indicated. PBS served as control. Supernatants were analyzed by TNF-α ELISA. Results are presented as mean ± SEM of three independent experiments run with duplicates. ANOVA with two Bonferroni-selected pairs of each individual ligand vs. ligand combination, as indicated. P** < 0.005, P*** < 0.001, n.s., not significant. [file 12974_2014_166_MOESM2_ESM.pdf]

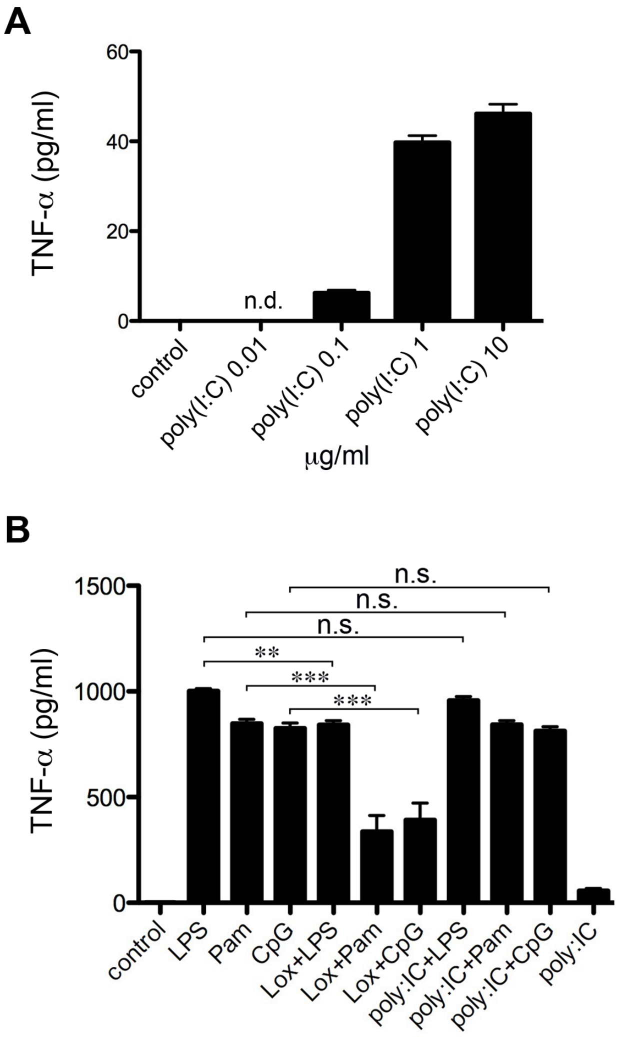

Supplementary Figure 2. The TLR3-specific ligand poly(I:C) does not suppress TNF-α secretion from microglia incubated with other TLR ligands. (**A**) Purified microglia from C57BL/6J mice were incubated for 6 h with various doses of poly(I:C), as indicated. PBS served as control. Supernatants were analyzed by TNF-α ELISA. Results are presented as mean ± SEM of 3 independent experiments run with duplicates. n.d., not detected. (**B**) Purified microglia from C57BL/6J mice were incubated for 6 h with LPS (100 ng/ml), Pam3CysSK4 (Pam, 100 ng/ml), CpG ODN (CpG, 1 μM), or poly(I:C) (1 μg/ml) alone or simultaneously with pairwise combinations of the ligands, as indicated. PBS served as control. Supernatants were analyzed by TNF-α ELISA. Results are presented as mean ± SEM of 3 independent experiments run with duplicates. ANOVA with

Bonferroni-selected pairs of each individual ligand *vs.* ligand combination, as indicated. $p^{**}<0.005$, $p^{***}<0.001$, n.s., not significant.
